# Stretchable Electronics Based on Laser Structured, Vapor Phase Polymerized PEDOT/Tosylate

**DOI:** 10.3390/polym12081654

**Published:** 2020-07-25

**Authors:** Zaid Aqrawe, Christian Boehler, Mahima Bansal, Simon J. O’Carroll, Maria Asplund, Darren Svirskis

**Affiliations:** 1 Department of Anatomy & Medical Imaging, School of Medical Sciences, University of Auckland, Auckland 1023, New Zealand; s.ocarroll@auckland.ac.nz; 2 Department of Microsystems Engineering (IMTEK) and BrainLinks-BrainTools Center, University of Freiburg, 79110 Freiburg, Germany; christian.boehler@imtek.de (C.B.); maria.asplund@imtek.uni-freiburg.de (M.A.); 3 School of Pharmacy, University of Auckland, Auckland 1023, New Zealand; m.bansal@auckland.ac.nz (M.B.); d.svirskis@auckland.ac.nz (D.S.); 4 Division of Nursing and Medical Technology, Luleå University of Technology, 971 87 Luleå, Sweden

**Keywords:** conducting polymers, vapor phase polymerization, PDMS, elastomeric

## Abstract

The fabrication of stretchable conductive material through vapor phase polymerization of poly(3,4-ethylenedioxythiophene) (PEDOT) is presented alongside a method to easily pattern these materials with nanosecond laser structuring. The devices were constructed from sheets of vapor phase polymerized PEDOT doped with tosylate on pre-stretched elastomeric substrates followed by laser structuring to achieve the desired geometrical shape. Devices were characterized for electrical conductivity, morphology, and electrical integrity in response to externally applied strain. Fabricated PEDOT sheets displayed a conductivity of 53.1 ± 1.2 S cm^−1^; clear buckling in the PEDOT microstructure was observed as a result of pre-stretching the underlying elastomeric substrate; and the final stretchable electronic devices were able to remain electrically conductive with up to 100% of externally applied strain. The described polymerization and fabrication steps achieve highly processable and patternable functional conductive polymer films, which are suitable for stretchable electronics due to their ability to withstand externally applied strains of up to 100%.

## 1. Introduction

Stretchable electronics have become a topic of focus for many academic and industrial research groups due to their ability to enable myriad emerging applications such as skin-mounted sensors, biosensors, soft robotics, and wearable displays [1,2]. The challenge in fabricating stretchable electronics resides within the inherent un-stretchable nature of commonly used metallic films, such as gold, which are known to fracture and fail electrically at strains of 1–2% [3] in both free-standing and polymer-supported states. Following approximately two decades of research, three main methods have been developed to achieve stretchable electronic materials: (i) engineering the geometry of rigid electrically conductive materials; (ii) the creation of a composite material with conductive fillers embedded into an elastomeric matrix; and (iii) the fabrication of intrinsically conductive materials [2]. Geometric engineering, employed by a number of research groups, has proven to impart stretchability onto rigid electrically conductive materials through the utilization of buckled or waved architectures. This method was initially described by Lacour et al. [4] and Jones et al. [5] in 2003 who discovered that deposition of thin layers (<100 nm) of chromium and gold onto pre-stretched elastomeric silicone substrates would create buckled structures in the metals nanostructure and enabled devices to withstand up to 26% strain without electrical failure, in a reproducible manner. The rationale behind these designs is that strain energy is dissipated through the straightening of buckled structures created through pre-stretching, rather than the electrical material itself. To build on this approach, interconnects can also be patterned into waved geometries on a macroscopic level to further contribute to the dissipation of strain upon stretching. In the literature, numerous waved geometries have been proposed, such as serpentine [6,7,8,9,10,11], horseshoe [12,13], kirigami [14], island [15], spiral [16,17], and fractal interconnects [18], which all serve to increase the maximum strain a device can withstand without failing electrically. To date, most of these devices which employ geometric engineering have been built with evaporated metallic films, such as gold and platinum (with their respective adhesion layers). The use of these materials is warranted due to their inertness, biocompatibility, and high conductivity. However, the difficulty that is associated with their functionalization for different applications and industries has drawn researchers to organic conductors, such as conductive polymers (CPs) [1,2,19]. CPs show great promise for stretchable electronics, due to their electronic tuneability by method of synthesis, ease of functionalization, and solution processability [20]—opening further avenues of deposition onto elastomeric substrates [21]. The most commonly employed CPs for stretchable electronics are cast from a commercially available aqueous dispersion of poly(3,4-ethylenedioxythiophene) (PEDOT) with poly(4-styrenesulfonate) (PSS) (PEDOT:PSS). Stretchable versions of PEDOT:PSS have been fabricated through geometric engineering [22], composite formation [2], and rendering the PEDOT:PSS to be intrinsically stretchable with additives [1]. To add to this rich body of literature, this article aims to arm readers with a novel approach of CP deposition for stretchable electronics though vapor phase polymerization (VPP), rather than solution casting of commercially available PEDOT:PSS films. VPP is a versatile method of achieving highly conductive, patternable [23,24] and thin CP films with easily tunable physical and electronic properties [21,24,25]. This communication aims to investigate the placement of VPP PEDOT films onto pre-stretched elastomeric substrates and the implications of this method on creating a stable stretchable electronic device. Furthermore, we introduce a method to laser structure VPP films in order to easily enable the patterning of multiple geometries of interest. The successful creation of a stable stretchable device which can be easily patterned and modified will open up avenues to a myriad of applications in fields such as bioelectronics, enabling the creation of microelectrode arrays that can study and help understand stretch-based injuries, for example traumatic brain injury or spinal cord injury. Furthermore, the versatility of this method allows for the ability to research various chemical additives and morphological structure and assess the impact these changes have on further optimization of this technology.

## 2. Materials and Methods

### 2.1. Vapor Phase Polymerization of PEDOT/Tosylate

The polymerization of PEDOT through vapor phase polymerization was carried out on a silicon wafer substrate. The wafer was first cleaned with isopropanol and then air dried using compressed air. An oxidant solution was prepared and comprised poly(ethylene glycol)-*block*-poly(propylene glycol)-*block*-poly(ethylene glycol) (PEG-PPG-PEG, 23% *w/w*, Sigma-Aldrich Chemie GmbH, Germany), iron (III) tosylate (FeTOS, 15.4% *w/w*, Sigma-Aldrich Chemie GmbH, Germany), and ethanol (61.5% *w/w*, Carl Roth GmbH + Co.KG, Karlsruhe, Germany). This oxidant solution was spun onto the silicon substrate at a speed of 1500 rpm for 20 s, with an acceleration of 500 rpm s^−1^, followed by heat curing at 70 °C for 60 s. These samples were then placed within a desiccator which contained 100 µL of 3,4-ethylenedioxythiophene (EDOT, Sigma-Aldrich Chemie GmbH, Germany) monomer in a petri-dish at its base. Air was evacuated from the desiccator using a vacuum pump followed by placement of the system in an oven with a temperature of 40 °C. The formation of PEDOT doped with tosylate (PEDOT/TOS) through VPP was allowed to occur under these conditions for 3 h [21]. At the end of this process, the samples were removed from the desiccator and rinsed with ethanol to wash away unreacted EDOT and residual FeTOS. Sequential polymerization steps obtained free-standing PEDOT/TOS films that could be transferred onto an elastomeric substrate, which meant additional layers polymerized over the previous PEDOT/TOS layer through repetition of the above process.

### 2.2. Transfer of PEDOT/TOS onto Elastomeric Substrate

Elastomeric substrates were made at dimensions of 50 mm × 50 mm × 1 mm (w × l × h) from poly(dimethyl siloxane) (PDMS, RTV MED 1000, APM Technica AG, Germany) using a custom made Teflon mold. PDMS was diluted with n-heptan (Carl Roth GmbH + Co.KG, Karlsruhe, Germany) at a ratio of 1:1 (v:v), poured into the mold and left to cure overnight in humid conditions. PDMS substrates were pre-stretched to varying degrees (0%, 40%, 60%, and 80%) of their original size using a custom-made stretching device and placed into a water bath. PEDOT/TOS films were removed from the silicon substrate in an ethanol bath; the free-standing film was then transferred to the water bath containing the pre-stretched PDMS at its base. A water bath was chosen for this transfer as the surface tension of the water was sufficient to hold the PEDOT/TOS films in a flat state on its surface. The stretching device containing pre-stretched PDMS was slowly raised out of the water; during this process, the overlying PEDOT/TOS film was picked up by the PDMS sample and “cast” onto the elastomeric substrate as it exited the water. The PDMS-PEDOT/TOS system was dried carefully with pressurized air and was then allowed to relax back to its non-stretched state.

### 2.3. Patterning of PEDOT/TOS on Elastomeric Substrate

A nanosecond laser (ACI Laser GmbH, Workstation Professional, Nohra, Germany) was used to pattern the conductive PEDOT/TOS into electronic tracts with a contact on each side to allow for resistance measurements while the system was put under varying degrees of strain. To efficiently etch PEDOT/TOS from the PDMS surface, the following parameters were used: laser power = 10% (0.4 Watts), speed = 100 mm s^−1^, frequency = 8 kHz, and pulse width = 3 µs. These parameters equate to a pulse power of 16.6 W and a pulse power density of 1.73 × 10^10^ Wm^−2^. Once the PEDOT/TOS was structured, the PDMS was cut into individual devices with dimensions of 7 mm × 20 mm, each containing one PEDOT/TOS tract (track dimensions of 500 µm × 2000 µm).

### 2.4. Conductivity Measurements

Surface electrical conductivity of the fabricated PEDOT/TOS films was determined at room temperature using a four-point linear probe set up with 1.0 mm tip separation. A benchtop multi-meter (Hewlett Packard 34401A, Boeblingen, Germany) was used to measure resistivity across 0.02″ tungsten probes (72T-J3, American Probe & Technologies Inc., Merced, CA, USA). A constant current was applied on the two outer electrodes, with the voltage drop across the two inner electrodes being measured. Measurements were made on three different locations across the film, and this was repeated on each subsequent polymerization layer. This process was done across three different PEDOT/TOS samples to obtain a triplicate conductivity value.

### 2.5. Strain vs. Resistivity Measurements

**Figure 1:**
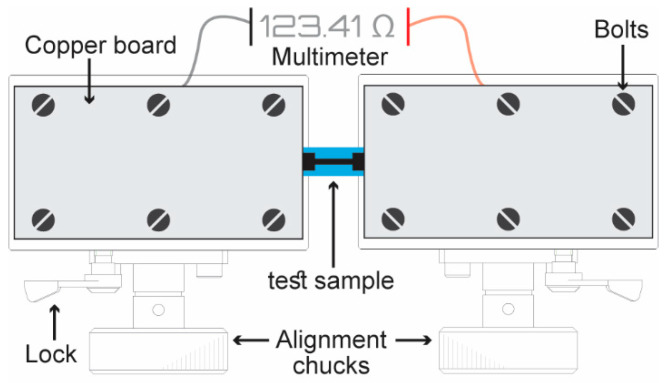
Stretch setup utilized which allows simultaneous resistivity measurements while the sample is put under strain.

To measure resistance of the PDMS-PEDOT/TOS device while under strain, a custom stretch system was assembled (Figure 1). This system consisted of two moveable alignment chucks secured side by side on a large base plate. The PDMS-PEDOT/TOS was positioned so that one contact was on one chuck and the other contact resided on the other chuck, with the middle (or tract section) freestanding between the two chucks. Copper boards were then placed on top of the PDMS-PEDOT/TOS contacts and tightly bolted down. The board served two purposes: (i) to provide an electrical connection; and (ii) to mechanically anchor the contacts onto the chucks. To stretch the sample, one chuck was locked into a fixed position and the other was slowly moved further away using an in-built positioning wheel. The stage was moved 0.25 mm each time and a two-electrode resistance measurement was carried out at each strain interval. The resistance measurement was taken for 10 s and the average value was calculated from this trace.

## 3. Results and Discussion

PEDOT/TOS was successfully polymerized onto a silicon wafer using a multi-layer approach via sequential vapor phase polymerization steps. Table 1 highlights the conductivity measured at each layer number using a four-point conductivity measurement. A steady increase can be noticed with the addition of each sequential layer, indicating inter-layer as well as intra-layer conductivity. This trend is consistent with other reports of layered PEDOT deposition [26] and is thought to arise due to the multiple parallel PEDOT-rich pathways, which form a more pronounced electrical network that increases conductivity [27]. To further improve conductivity, conditions to control the VPP process temperature and pressure more accurately can be implemented, such as utilization of a vacuum oven. Previous experiments carried out in a vacuum oven achieved a conductivity of 1840 ± 50 S cm^−1^ for one layer of PEDOT/TOS [21].

**Figure 2:**
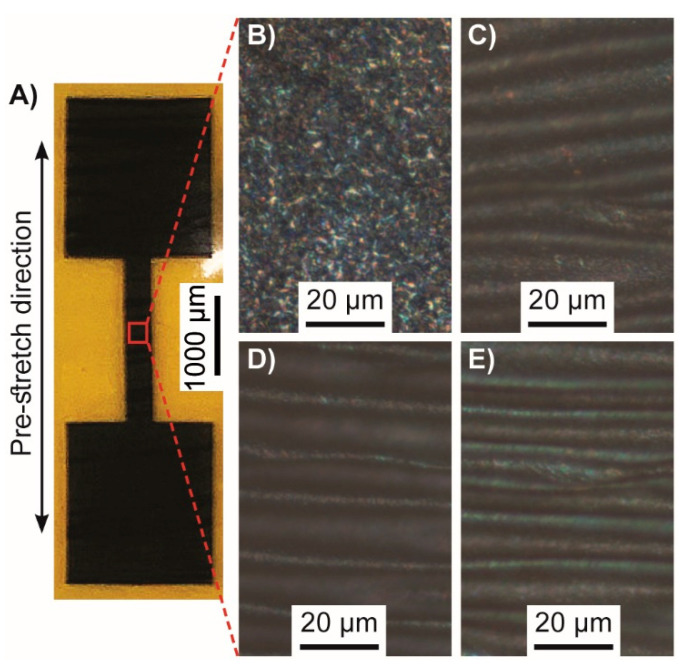
(**A**) Final device design with two contact pads joined by a 500 µm wide by 2000 µm long track. The device shown illustrates a laser structured PEDOT/TOS film on PDMS which was pre-stretched by 80%. The device is clear, however, here it was imaged on a Kapton substrate, giving it a yellow color. The close-up images show the structure of PEDOT/TOS on PDMS which had: (**B**) 0% pre-stretch; (**C**) 40% pre-stretch; (**D**) 60% pre-stretch; and (**E**) 80% pre-stretch. Clear buckled structures can be seen in PEDOT/TOS films which were placed onto pre-stretched PDMS that was then allowed to relax.

At the third sequential polymerization layer, the PEDOT/TOS had enough mechanical stability to peel off from the silicon substrate and form a free-standing film in water or ethanol and this was taken as the number of layers employed for the stretchable electronic devices. In a previous publication, the thickness of five PEDOT/TOS layers prepared by a similar VPP process was determined to be 1.66 ± 0.06 μm, with an unremarkable surface morphology; therefore, it is estimated that the total thickness of these films resides around 1 µm [21]. PEDOT/TOS films were successfully transferred to pre-stretched PDMS substrates and structured to the desired pattern using a nano-second laser (Figure 2A). When examining the microstructure of these films, buckling can clearly be seen perpendicular to the stretch direction and is as a result of laying down the conductive PEDOT/TOS films onto pre-stretched PDMS that is then allowed to relax (Figure 2C–E).

**Figure 3:**
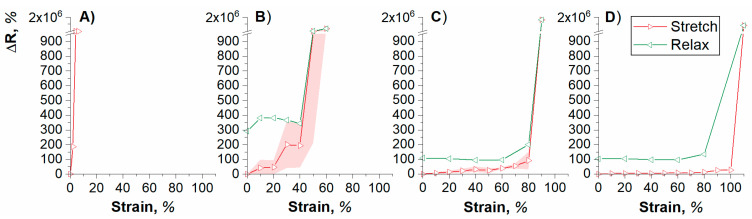
Strain vs. change in resistance profile of PDMS-PEDOT/TOS system under: (**A**) 0% pre-stretch; (**B**) 40% pre-stretch; (**C**) 60% pre-stretch; and (**D**) 80% pre-stretch. The graphs show both the stretching (red) and relaxing (green) cycles and the shaded areas indicate standard deviation (n = 3, plotted as the average ± SD).

The strain vs. change in resistance profile (ΔR) of varying pre-stretched PEDOT/TOS samples is displayed in Figure 3. A sharp increase in ΔR signified electrical disruption at the corresponding applied external strain. Analysis of this graph highlights the dependency of degree of pre-stretch on the maximum strain load that can be handled. In other words, the higher is the pre-stretch, the more functional strain the device can accommodate when in use. At 0% pre-stretch, the PEDOT films fail electrically after the first incremental strain step of 2%, demonstrating the low intrinsic stretchability of the PEDOT/TOS material. Strategies to improve the intrinsic stretchability of PEDOT:PSS films have been highlighted in previous studies and could also be implemented in future works to further improve the performance of these PEDOT/TOS devices. The process of blending PEDOT:PSS with additives such as small molecule plasticizers, e.g., glycerol [28] and ionic liquids [29], has been reported and allows the PEDOT films to be stretched up to 50% without any detrimental effect on electrical properties [1]. In a similar fashion, the use of polymer additives such as poly(ethylene glycol) (PEG) [30], poly(ethylene oxide) (PEO) [30], poly(ethylene glycol)-block-poly(propylene glycol)-block-poly(ethylene glycol) triblock copolymer (PEO-PPO-PEO) [31], and poly(vinyl alcohol) [1] in the PEDOT matrix supported strain values of up to 100%; however, one disadvantage is the decrease in electronic conductivity that arises upon addition of insulating polymeric materials. It should be noted that the current mixture used for VPP polymerization contains PEG-PPG-PEG, which is also theorized to impart some stretchable properties to the CP film, and further optimization of this mixture could provide more favorable intrinsic stretchability in future studies. Increasing the pre-stretch to 20% yielded a higher tolerance to externally applied strain, where the films failed at an external strain of above 20%. At higher pre-stretches of 60% and 80%, the films withstood externally applied strains of 80% and 100%, respectively, before electrical disruption. An interesting observation for all pre-stretched samples was the return of an electrical path once the samples were allowed to relax after a sharp increase in resistance, highlighting the presence of compensatory conductive pathways. It should be noted that there is a mismatch in resistance of these “relaxed” devices when compared to pristine ones and this is likely to occur as a result of conductive path breakages during the stretch cycle; nonetheless, these data highlight the ability of these materials to maintain electrical conductivity even after their respective maximum strain limits are reached. It is again apparent that PEDOT/TOS subjected to greater pre-stretch (>60%) produced a more stable system through observation of the relax phase which shows that the resistance returns closer to pristine levels when compared to a pre-stretch of 40%. This may be explained by the way strain is dissipated in CPs subjected to greater pre-stretch, whereby breakages or disconnects occur in different ways. These data indicate the ability of the introduced buckled structures (Figure 2E) to successfully dissipate the strain energy and create functioning stretchable electronics through the utilization of VPP based PEDOT/TOS. It should be noted that, during these stretch/relax processes, the PEDOT/TOS did not delaminate from the underlying PDMS substrate in any of the samples tested indicating good adhesion between the two surfaces. Future in-depth mechanical testing is needed to quantitively assess the strength of this interface, such as the tape test to quantify strength of adhesion [32].

## 4. Conclusions

The applicability of vapor phase polymerized PEDOT films for stretchable electronics is demonstrated, showing that these devices can withstand up to 100% strain under the right fabrication conditions. In addition, electrical pathways were partially restored upon relaxation of films, even after the maximum strain was surpassed. Further optimization of the polymerization process, and the introduction of additives to improve PEDOT film stretchability, are likely to further improve the performance of these devices. The ease of patterning through laser structuring was demonstrated and allows for many geometries to be tested using these techniques. It should be noted that, in our study, the conducting polymer is insulated only on one side and is fully exposed to the top. In certain applications, such as bioelectronics, a top insulating layer is needed to define electrode area and to protect the conducting polymer from factors such as humidity. Although not tested here, we believe this insulating layer could further help to mechanically stabilize the PEDOT layer as long as the bottom and top silicone layers could be put under equal tension. The myriad possibilities offered by vapor phase polymerization, and the ease of patterning through laser structuring described in this communication, opens doors for new research avenues in stretchable electronic devices.

## Figures and Tables

**Table 1:** Conductivity associated with number of PEDOT/TOS layers.

Layer Number	Conductivity (S cm^−1^)
1	37.7 ± 1.4
2	43.2 ± 2
3	53.1 ± 1.2
